# Health-related quality of life among critically ill patients after discharge from the ICU—A systematic review protocol

**DOI:** 10.1371/journal.pone.0278800

**Published:** 2023-08-18

**Authors:** Yao Li, Dan Fang, Qiao Wu

**Affiliations:** 1 Department of Intensive Care Unit, Chengdu Fifth People’s Hospital, Sichuan, People’s Republic of China; 2 Department of Orthopedics, Sichuan Academy of Medical Sciences, Sichuan Provincial People’s Hospital, Sichuan, People’s Republic of China; Xiamen University - Malaysia Campus: Xiamen University - Malaysia, MALAYSIA

## Abstract

The impact of critical illness on patients is profound, resulting in physical, mental, and social consequences and poor health-related quality of life (HRQOL). Several studies investigated HRQOL among patients discharged from the intensive care unit (ICU). However, few systematic reviews cover studies conducted in the last decade or using valid instruments for measuring HRQOL in general ICU survivor populations. Herein, we conduct a systematic review of these studies that followed PRISMA guidelines. We will search PubMed, Web of Science, CINAHL (Cumulative Index to Nursing and Allied Health Literature), Cochrane Library, and Open Grey for papers. We will search for articles reporting the HRQOL of ICU survivors that were written in English and published from 01 January 2012 onward from the date of this protocol’s publication. We will also extract HRQOL data and analyze associate factors. The risk of bias will be measured with a standard quality assessment tool. The strength of the results will depend on the number of studies and the consistency of their results.

**Trial registration number:** PROSPERO CRD 42022304279.

## Introduction

### Rationale

With improved intensive care medicine, most critically ill patients survive critical conditions and move out of the intensive care unit (ICU) [1, 2]. According to the Society of Critical Care Medicine statistics, over five million patients are admitted to the ICU annually in the USA. Over 80% of patients survive after critical illness in the ICU [3]. These patients are called ICU survivors and benefit from advanced intensive care while suffering the sequela of critical illness. Over half of ICU survivors suffer from physical, mental, and cognitive impairments in what is known as post-ICU syndrome (PICS) [4]. In addition, these patients may experience poor health-related quality of life (HRQOL) for three months to ten years after discharge from the ICU [5–9]. Patients with critical illnesses admitted to the ICU commonly have poor HRQOL compared with the general population [9]. Health-related quality of life is the perception of how patients’ quality of life is affected by their critical illness experience and being in the ICU [10]. Poor HRQOL among ICU survivors is a significant concern linked to higher mortality, financial burden, and family caregiver burden [11–13]. Indeed, survivors with better HRQOL generally have less anxiety, depression, and Post-traumatic stress disorder (PTSD) [14–16]. Over the past several decades, poor HRQOL among ICU survivors has received increasing attention from researchers, leading to a growth in HRQOL research.

Elliott et al. (1999) reviewed 31 studies, but only some used valid instruments [17]. Dowdy et al. (2005) examined 21 studies that included 7320 patients and found that ICU survivors suffered poor HRQOL and that age and severity of illness were associated with HRQOL [18]; however, they did not analyze other factors. Hauge and colleagues conducted a systematic review in a subgroup of patients who suffered from sepsis, major trauma, or severe burn injuries. They found that HRQOL among these groups was similar [19]. However, these outcomes may be less generalizable when applied to general ICU survivors’ health status [19]. A systematic review by Gerth et al. (2019) found 48 studies from 2000 to 2015. They showed that ICU survivors had poor HRQOL compared to the sex-matched population and that overall, HRQOL improved with time [8]. A published by Ariyo et al. in 2021 analyzed 18 studies that included 2090 elderly ICU survivors that were followed over 3 to 100 months [20]. Elderly ICU survivors had worse quality of life than younger survivors [20]. However, most reviews contained studies over 20 years old that may not accurately reflect HRQOL among ICU survivors today [21]. Previous systematic reviews primarily included studies published in Western countries [8, 17, 18, 21]. In recent years, HRQOL among ICU survivors in Asian countries has received increasing attention [22, 23]. Published reviews to date may not provide a comprehensive description of HRQOL among ICU survivors. Generic instruments for assessing HRQOL include tools within the short-form health survey (SF) and EqurQol (EQ) family, including SF 36, SF 12, SF 20, EQ-5D-3L and EQ-5D-5L [24, 25]. The SF 36 has eight dimensions that reflect HRQOL: physical function, role-physical, social function, body pain, general health, vitality, role-emotion and mental health [24]. The EQ-5D is a nonspecific disease instrument that assesses mobility, self-care, usual activities, pain/discomfort, and anxiety/depression [25]. The SF 36 (version 1 and version 2) and the EQ-5D-5L have been used extensively in critically ill patient populations [9]. Therefore, this review will analyze studies that used valid instruments: SF 36 and EQ-5D. In addition, we will examine studies in which the SF 36 and EQ-5D-5L tools were administered to ICU survivors and used to measure HRQOL over time.

Our review will include only studies published from January 2012 onward, focusing on all types of ICU admission rather than specific events. We will search for studies that measure HRQOL in general ICU admission populations and keep only those that used reliable instruments. We aim to examine the factors that may influence HRQOL, such as length of ICU stay, age, and the severity of illness [7, 9, 22, 26]. Compared to past studies, the follow-up duration has been extended. Some researchers followed ICU survivors after they were discharged from the ICU for up to ten years (5). Our primary objective is to determine whether HRQOL improves with time. The second objective is identifying the factors influencing HRQOL in the follow-up period. We will guide the research to provide comprehensive, detailed, descriptive information concerning long-term outcomes for general ICU survivors.

## Method

### Ethics statement

No patients were involved in this study.

### Design

This protocol follows (PRISMA-P) guidelines. We will systematically review the literature published in peer-reviewed journals related to HRQOL among patients discharged from the ICU. The articles we will examine were published in English from January 2012 and onward from the date of this protocol’s publication.

### Information sources

A literature search will be conducted following the publication of this study protocol. We will search PubMed, Web of Science, Cochrane Library, CINAHL, and OpenGrey for studies written in English that report the HRQOL of ICU survivors from January 2012 and onward from the date of the protocol’s publication.

### Search strategy

Our search strategy will be as follows: (“critical care” OR “critical illness” OR “Intensive care unit”) AND (“Health-related quality of life” OR “quality of life” OR “health status indicator” OR “QQL” OR “long-term outcome”). We will review the reference list of primary studies identified in our search and the reference lists of relevant previously published reviews on HRQOL in ICU survivors.

### Eligibility criteria

We will identify prospective, retrospective, cross-sectional studies identified by the search terms and published within the review time frame will be reviewed for inclusion.

**Fig 1:**
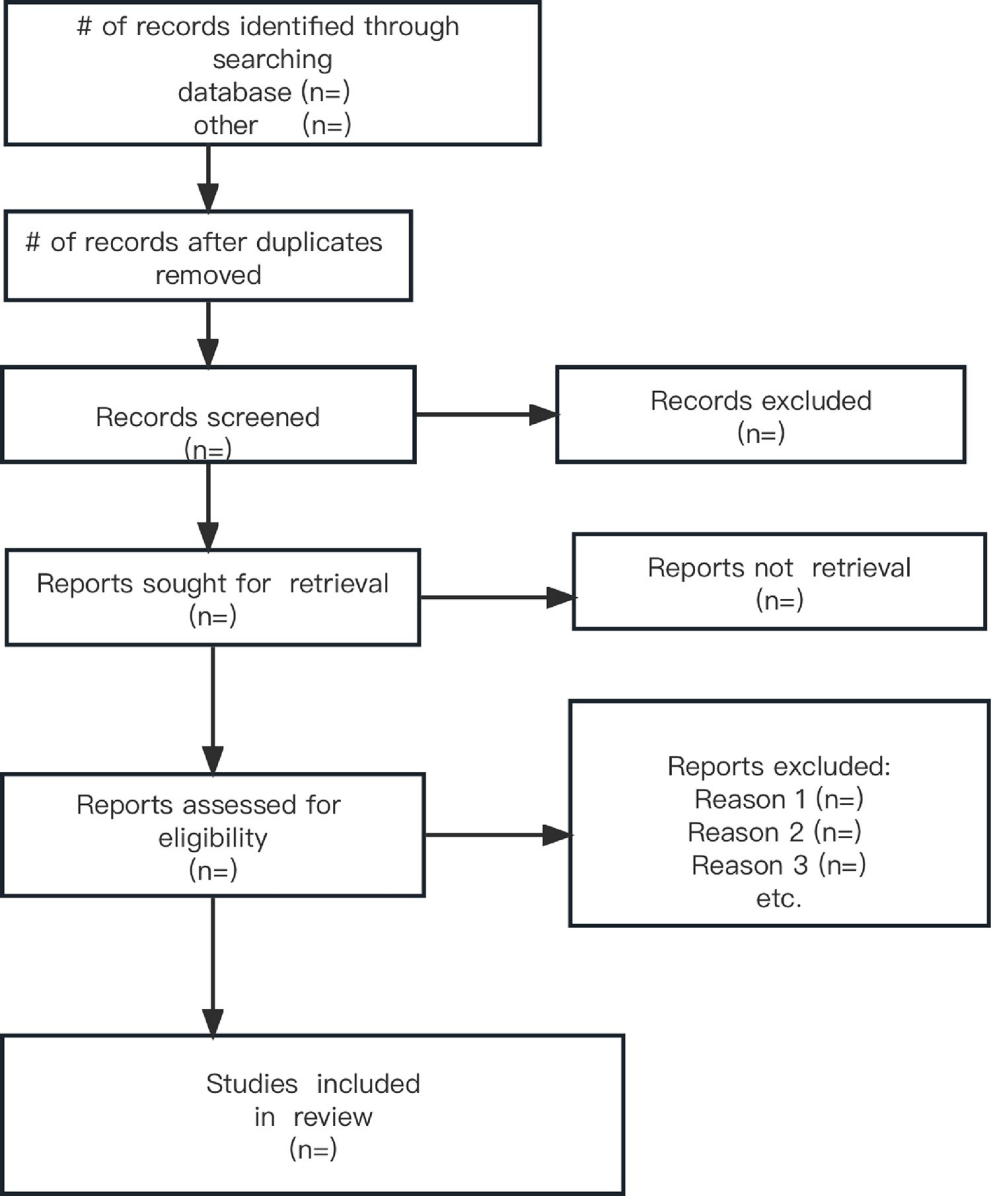
This is the selection diagram.

A flow diagram adapted from the PRISMA guidelines [27] will outline which studies were included in each stage of the systematic review (Fig 1).

### Study inclusion criteria

Adult ICU survivors over the age of 18.

Original research

Quality of life assessed at least one month after discharge from the ICU.

Measurement and reporting of specific QOL domains at baseline and follow-up

Compared HRQOL with an age-adjusted population.

General ICU settings (medical/surgical/mixed)

Valid measurement of the SF 36 (Version one/Version two) and EQ-5D-5L

English language

### Exclusion criteria

The study only targets specific events, for example, postcardiac arrest, COVID-19, and ECMO (Extracorporeal Membrane Oxygenation) patients.

Specific age populations were studied (we will target general ICU survivors, as the exact age group might affect the results)

The study included only specific diseases or one disease; for example, trauma (we will focus on all types of ICU admission as we want to determine the impact on HRQOL of ICU treatment or ICU experience particularly, rather than the experience of a specific disease)

Experimental studies without a control group

We will choose the study with the most prolonged follow-up period if the study presents the same patient data set as another study.

### Data management

We will store identified citations and electronic text in the reference manager program Endnote 20.

### Selection process

Two researchers (DF and QW) will undertake the screening process. They will search for titles and abstracts of articles and will independently screen search results for potential studies. They will follow PRISMA-P guidelines. The two researchers will each remove abstracts that do not match the eligibility criteria. In the event of a disagreement regarding removing an abstract, the two researchers will discuss to reach a consensus. If necessary, a third researcher (YL) will be consulted to resolve any remaining disagreements.

The two researchers will independently conduct a final screening process of the full-text articles to select those that meet our eligibility criteria. For any article excluded, the reason for exclusion will be documented. The papers included in each stage of the systematic review will be outlined in a diagram adapted from the PRISMA statement (Fig 1).

### Data extraction method

The data extraction process will be conducted after this protocol is published. Two review authors (DF and QW) will extract data from eligible studies. Duplicated studies will be removed. Data extractions will be included if a consensus can be reached. We will extract data from the full-text article and online supplementary data. All the data will be organized in a data extraction table. If information from the studies is unclear, we will contact the authors to clarify and exclude ambiguous data if we cannot reach the author.

We will extract the following information:

Citations

Study country of origin

Study design

Year of publication

Type of ICU

Patient type: general/sepsis/respiratory

The number of patients screened.

The total number of patients who were alive at the start of the study.

Successful follow-up rate

Follow-up time points

Proxy ICU HRQOL assessment

Whether data on HRQOL were gleaned from studies and online resources

Whether HRQOL is relative to the age-adjusted average population

Previous health/preexisting disease

The trend in HRQOL post-discharge

Factors affecting HRQOL post-discharge.

The primary outcome is HRQOL among ICU survivors. Our second objective is to examine changes in HRQOL over months to years after patients’ discharge from the ICU. The secondary outcome is determining the factors influencing HRQOL during the follow-up period.

### Synthesis of data and data analysis method

All the data from eligible studies will be downloaded into Microsoft Excel with exact information, including citations, study country of origin, study design, and type of ICU. In tabular form, the specific outcome was HRQOL, and we will describe the HRQOL among ICU survivors. We will describe secondary outcomes (factors that affect HRQOL, length of follow-up) in tabular or graphical form.

### Bias assessment

We will assess the papers for applicability and bias using the Standard Quality Assessment tool developed by Kmet and colleagues [28], a standard quality assessment tool for primary studies in which RCTs (randomised controlled trials) were not performed. There are 14 items to score in this assessment that indicate the degree to which studies meet the quality criteria.

### Strengths and limitations of this study

This study will focus on general ICU survivors to ensure our results are generalisable.

We will focus on recently published studies, allowing us to observe whether the HRQOL has changed compared to earlier studies. Due to the heterogeneity of study designs and populations, we will not conduct a meta-analysis of the study findings or assess meta-biases statistically. The number of studies available and the study results’ consistency will determine the conclusions’ strength.

## Supporting information

S1 Checklist This is the PRISMA-P 2015 checklist. (DOC)
